# Supplementary material for: Uncertainty-Aware First-Principles Exploration of Chemical Reaction Networks
Source: J Phys Chem A. 2024 May 24;128(22):4532–47. doi: 10.1021/acs.jpca.3c08386 (PMC11163430; doi:10.1021/acs.jpca.3c08386)
Supplement: Supplementary file 1 — jp3c08386_si_001.pdf [file jp3c08386_si_001.pdf]

Uncertainty-aware First-principles Exploration of Chemical Reaction Networks

Moritz Bensberg[1] and Markus Reiher[2]

ETH Zürich, Department of Chemistry and Applied Biosciences, Vladimir-Prelog-Weg 2, 8093 Zürich, Switzerland

Supporting Information

Date: 09.04.2024

[1]ORCID: 0000-0002-3479-4772

[2]email: markus.reiher@phys.chem.ethz.ch; ORCID: 0000-0002-9508-1565

1 Convergence of the Flux-based Sensitivity Analysis Prescreening

To analyze the effect of the flux-based prescreening, which restricts the parameters varied during the Morris Sensitivity analysis, we ran the Morris sensitivity analysis for the microkinetic model of the converged exploration based on the local one-at-a-time sensitivities and the Eschenmoser–Claisen rearrangement of furfuryl alcohol. We carried out these microkinetic modeling simulations for truncation thresholds $\tau_{\text{flux}}^{\text{sens}}$ between $0.0\ \text{mol}\,\text{L}^{-1}$ and $1 \cdot 10^{-7}\ \text{mol}\,\text{L}^{-1}$. The effect of the flux-based truncation on the predicted standard deviations of the concentrations σ is illustrated in Tab. SI. We list the maximum change [Abs(max $\Delta\sigma$)] of the standard deviation for each threshold compared to $\tau_{\text{flux}}^{\text{sens}} = 0.0\ \text{mol}\,\text{L}^{-1}$, the maximum relative change [Rel(max $\Delta\sigma/\sigma_0$)] for every standard deviation larger than $0.05\ \text{mol}\,\text{L}^{-1}$ (*i.e.*, standard deviations similar to the exploration thresholds τ_{flux} and τ_{max}), as well as the values of the reference standard deviations (σ_0) associated with these maximum differences. As is clear from the table, the effect on the standard deviations is significant for all non-zero thresholds as it is above $0.05\ \text{mol}\,\text{L}^{-1}$. However, these large changes affect always the same species with a large standard deviation of $0.375\ \text{mol}\,\text{L}^{-1}$. Therefore, the parameter restriction should not affect the exploration conditions (see Eqs. (13) and (14) in the main text) that depend on the standard deviation significantly.

2 Convergence of the Morris Sensitivity Analysis with the Sample Size

To investigate the convergence of our sensitivity measures with respect to the sample size for the Morris sensitivity analysis, we performed the Morris sensitivity analysis with

Table SI: Effect of the flux-based parameter restriction for the sensitivity analysis on the standard deviation for the concentrations.

$\tau_{\text{flux}}^{\text{sens}}/(\text{mol}\,\text{L}^{-1})$	$1 \cdot 10^{-10}$	$1 \cdot 10^{-9}$	$1 \cdot 10^{-8}$	$1 \cdot 10^{-7}$
Abs(max $\Delta\sigma$)	0.051	0.089	0.057	0.096
Rel(max $\Delta\sigma/\sigma_0$)	0.136	0.479	0.152	0.256
σ_0(Abs.)	0.375	0.375	0.375	0.375
σ_0(Rel.)	0.375	0.109	0.375	0.375

sample sizes of $N = 10$ to $N = 30$ for the microkinetic model of the Eschenmose–Claisen rearrangement explored with the local one-at-a-time sensitivity-based exploration. For these microkinetic modeling simulations, we monitored which parameters would be selected for refinement according to the refinement conditions assumed in this work, *i.e.*, $\mu_i^{*\max} > 5 \cdot 10^{-2}$ mol L^{-1}.

The number of parameters considered for refinement, as well as the number of parameters not selected for refinement compared to a sample size of $N = 30$, are given in Tab. SII. The Morris sensitivity analysis is effective in identifying most of the important parameters already at a low sample size of $N = 15$. However, there are still 3 parameters not selected for refinement for $N = 25$, which would be selected for $N = 30$. Because the number of parameters in the microkinetic modeling simulations can quickly become very large (more than 4000 in the largest simulation in this work), we aimed at using as many samples as possible without having an exceedingly large runtime of the sensitivity analysis.

Table SII: Number of parameters considered for the refinement as a function of the sample size in the Morris sensitivity analysis and the number of parameters missing in the refinement compared to a sample size of $N = 30$.

N	10	15	20	25	30
Parameters refined	18	17	18	24	20
Parameters missing vs. $N = 30$	5	3	3	3	0
